# Delayed treatment of tuberculosis patients in rural areas of Yogyakarta province, Indonesia

**DOI:** 10.1186/1471-2458-8-393

**Published:** 2008-11-26

**Authors:** Yodi Mahendradhata, Bobby M Syahrizal, Adi Utarini

**Affiliations:** 1 Department of Public Health, Faculty of Medicine, Gadjah Mada University, Yogyakarta, Indonesia; 2 FIDELIS project, Faculty of Medicine, Gadjah Mada University, Yogyakarta, Indonesia; 3 Epidemiology and Disease Control Unit, Public Health Department, Institute of Tropical Medicine, Antwerp, Belgium

## Abstract

**Background:**

In year 2000, the entire population in Indonesia was 201 million and 57.6 percent of that was living in rural areas. This paper reports analyses that address to what extent the rural structure influence the way TB patients seek care prior to diagnosis by a DOTS facility.

**Methods:**

We documented healthcare utilization pattern of smear positive TB patients prior to diagnosis and treatment by DOTS services (health centre, chest clinic, public and private hospital) in Yogyakarta province. We calculated the delay in treatment as the number of weeks between the onset of symptoms and the start of DOTS treatment. Statistical analysis was carried out with Epi Info version 3.3 (October 5, 2004).

**Results:**

The only factor which was significantly associated with total delay was urban-rural setting (p = < 0.0001). The median total delay for TB patients in urban districts was 8 (1^st ^Quartile = 4; 3^rd ^Quartile = 12) weeks compared to 12 (1^st ^Quartile = 7; 3^rd ^Quartile = 23) weeks for patients in rural districts. Multivariate analysis suggested no confounding between individual factors and urban-rural setting remained as the main factor for total delay (p = < 0.0001). Primary health centre was the first choice provider for most (38.7%) of these TB patients. Urban-rural setting was also the only factor which was significantly associated with choice of first provider (p = 0.03).

**Conclusion:**

Improving access to DOTS services in rural areas is an area of vital importance in aiming to make progress toward achieving TB control targets in Indonesia.

## Introduction

Indonesia ranks third in the world for TB burden [1]. There were 245 new TB cases per 100,000 persons and 110 new sputum smear-positive cases per 100,000 in 2004. Although the global target for treatment success (85%) has been achieved in the country since the year 2000, progress toward the case detection target (70%) has been lagging behind, reaching 53% in 2004. There are many possible reasons underlying this low case detection rate, including socio-demographic factors and health service utilization pattern. Therefore better understanding of socio-demographic factors and health service utilization pattern could help identify reasons for low or delayed accessing of DOTS (Directly Observed Treatment, Short-course) services in Indonesia as well as potential interventions.

As with most infectious diseases, TB is not randomly distributed [2]; it thrives in specific groups and under specific conditions in association with identified and unidentified factors that confer vulnerability to the disease including poverty [2-5]. Rural residence can be a marker for poverty [6], thus vulnerability to TB as well. In year 2000, the share of people below the poverty line in Indonesia (per capita monthly household incomes below US$ 11 for urban areas; below US$9 for rural areas) was 22.4 percent in rural areas, while it was 14.6 percent in urban areas [7]. In that year, the entire population in Indonesia was 201 million and 57.6 percent of that was living in rural areas.

Population below poverty line was approximately two times larger in rural areas than in urban areas, and poverty is a major problem in Indonesian rural areas. Moreover, most people in rural Indonesia have to pay their medical expenses on an out-of-pocket basis, which could potentially constitute a significant financial barrier to access TB treatment.

The National Tuberculosis Control Programme (NTP) is entering a phase of rapid and comprehensive acceleration of its TB activities thanks to a substantial increase in funding mainly through grants from the Global Fund to Fight AIDS, TB and Malaria [8]. With a fully-funded budget, many opportunities are being taken to expand coverage of the DOTS strategy. Comparison of barriers to DOTS between rural and urban areas will help to expand DOTS expansion, boost case detection and shorten total delay.

The special province of Yogyakarta is located in the central Southern part of Java. The provincial administration is divided into two urban and three rural districts with a total population of 3.2 million inhabitants in an area of 3,185 km square, which implies a population density of 1055 per km square. About 44,49% of the population are rural residence. Between 1999 and 2002, poverty in the urban areas decreased from 26.90% to 16.90%. In the same period, poverty in rural areas poverty increased from 23.10% to 25.10%.

The province's primary health care network consists of approximately 650 private practitioners and 117 community health centres. These first line services are backed up by 9 public hospitals and 24 private hospitals (including maternity and mental hospitals) which are mostly located in the urban areas.

There are many activities to strengthen TB services in Yogyakarta. Recognizing the need to involve hospitals, which manage a large proportion of TB cases in Indonesia, KNCV Tuberculosis Foundation and the University of Alabama support a "Hospital-DOTS Linkage (HDL)" project in Yogyakarta since 2000. The project is a joint collaboration of the National Tuberculosis Programme (NTP) and Persatuan Rumah Sakit Indonesia (PERSI, the Indonesian Hospital Association) to expand DOTS coverage to the hospitals. More recently, the HDL strategy was used to develop an initiative to involve private practitioners in TB control with support from Fund for Innovative DOTS Expansion through Local Innovative Solutions (FIDELIS).

This paper describes to what extent the rural structure influence TB control programme performance and how do TB patients seek care prior to diagnosis by a DOTS facility in rural areas of Yogyakarta province. We shall argue that the challenge for TB control in these rural areas is substantial and that action thus must be taken to improve access to DOTS services for the large masses of people who still inhabit rural areas.

## Methods

We introduced into all DOTS services (health centres, chest clinics, public and private hospitals) in the province the Limited Access (LA) forms developed by FIDELIS as part of the project monitoring and evaluation system. The LA form documented demographic characteristics, type and duration of TB symptoms (persistent cough, bloody cough, chest discomfort, weight loss, fevers) and healthcare utilization pattern of smear positive TB patients prior to diagnosis and treatment by DOTS services. Health workers of the DOTS services were trained to administer the LA forms and complete them as soon as smear-positive TB diagnosis has been made. The completed forms were collected on a monthly basis by field supervisors. The forms were checked for completeness. In the case of forms identified to be incomplete, the health worker or supervisor had to do a second interview as soon as possible to remediate. The completed data was entered into an electronic database in the project secretariat.

We refined the data and checked for consistency prior to analysis. We calculated the delay in treatment as the number of weeks between the onset of symptoms and the start of DOTS treatment. In univariate analysis, differences between proportions were tested with Chi-square and differences between means were tested with T-test. Linear regression was performed to identify independent determinants of total delay. Statistical analysis was carried out with Epi Info version 3.3 (October 5, 2004).

The study was approved by the ethical review committee of the Faculty of Medicine, Gadjah Mada University, Indonesia.

## Results

A total of 1116 patients were registered as smear positive cases in Yogyakarta province during May 2004–February 2005; we give results for 421 (37.7%) about whom data on the time between onset of TB symptoms and treatment initiation had been recorded.

Most (54.6%) of the TB patients in the FIDELIS dataset was aged between 25–54 years old. There were slightly more males (57.7%) than females (42.3%). There were also slightly more patients coming from urban districts (52.7%) than rural areas (47.3%). Median total delay was 10 (1^st ^Quartile = 5; 3^rd ^Quartile = 17) weeks. Univariate analysis (Table 1.) showed that the only factor which was significantly associated with total delay was urban-rural setting (p < 0.0001). The median total delay for TB patients in urban districts was 8 (1^st ^Quartile = 4; 3^rd ^Quartile = 12) weeks compared to 12 (1^st ^Quartile = 7; 3^rd ^Quartile = 23) weeks for patients in rural districts. Age, gender, first symptom, and first provider were not associated with total delay.

**Table 1:** Characteristics of smear-positive TB patients and total delay in Yogyakarta, Indonesia, May 2004–February 2005

**Characteristic**	**n**	**%**	**Mean**	**SD**	**Median**	**1^st ^Quartile**	**3^rd ^Quartile**	**P**
*Age*								
15–24	80	19.0	10.29	7.99	8.0	4.0	12.0	
25–54	230	54.6	13.78	11.77	11.0	5.0	19.0	0.069
≥ 55	111	26.4	14.30	12.19	11.0	6.0	18.0	
								
*Gender*								
Male	243	57.7	13.30	11.38	10.0	5.0	17.0	
Female	178	42.3	13.20	11.25	10.0	6.0	16.0	0.982
								
*Setting*								
Urban	222	52.7	10.54	9.31	8.0	4.0	12.0	
Rural	199	47.3	15.69	12.37	12.0	7.0	23.0	< 0.0001
								
*First symptom*								
Cough	335	79.6	12.81	10.76	10.0	5.0	16.0	
Others	86	20.4	15.00	13.18	9.0	6.0	20.0	0.401
								
*First provider visited*								
Private Practitioner	115	27.3	14.15	11.64	11.0	6.0	21.0	
Health Centre	163	38.7	12.42	10.13	9.0	5.0	18.0	0.628
Hospital	75	17.8	14.20	13.77	8.0	4.0	17.0	
Chest Clinic	68	16.2	12.70	10.50	11.0	5.0	16.0	
								
All	421	100.0	13.25	11.31	10.0	5.0	17.0	

Linear regression was performed to examine the effect of potential confounders on the relationship observed. This analysis suggested no confounding between individual factors and urban-rural setting remained as the main factor for total delay at significance level similar to what was observed in the univariate analysis (p =< 0.0001). Age became significant in this multivariate analysis with shorter total delay among those within the age group of 25–54 years old (p = 0.01) and ≥ 55 years old (p = 0.02).

Primary health centre was the first choice provider for most (38.7%) of these TB patients (Table 2.). Urban-rural setting was also the only factor which was significantly associated with choice of first provider (p = 0.03). Private practitioner (PP) was ranked second (27.3%) as first choice provider with more rural patients (31.1%) opting for PPs compared to patients in urban settings (27.5%).

**Table 2:** Characteristics of smear-positive TB patients and choice of first provider in Yogyakarta, Indonesia, May 2004–February 2005

	First choice provider										
				
Characteristic	Private Practitioner		Health Centre		Hospital		Chest Clinic		Total		P
		
	N	%	n	%	n	%	n	%	n	%	
*Age*											
15–24	24	30.0	31	38.8	12	15.0	13	16.3	80	100.0	
25–54	67	29.1	85	37.0	44	19.1	34	14.8	230	100.0	0.707
≥ 55	24	21.6	47	42.3	19	17.1	21	18.9	111	100.0	
											
*Gender*											
Male	67	27.6	93	38.3	43	17.7	40	16.5	243	100.0	
Female	48	27.0	70	39.3	32	18.0	28	15.7	178	100.0	0.994
											
*Setting*											
Urban	46	23.1	78	39.2	46	23.1	29	14.6	222	100.0	
Rural	69	31.1	85	38.3	29	13.1	39	17.6	199	100.0	0.029
											
*Initial symptom*											
Cough	92	27.5	125	37.3	54	16.1	64	19.1	335	100.0	
Others	23	26.7	38	44.2	21	24.4	4	4.7	86	100.0	0.006
											
All	115	27.3	163	38.7	75	17.8	68	16.2	421	100.0	

## Discussion

Our results showed that TB patients in rural areas are less likely to first seek care at DOTS facilities and took significantly much longer time to arrive at DOTS facilities. This highlights issues of care seeking behaviour and ultimately access to DOTS services in rural areas. There is a growing body of evidence on how such challenge can be addressed, including: village community outreach [9]; involvement of rural private practitioners [10,11]; community empowerment [12]; poverty reduction schemes [5]; increased state investment in basic health [13].

The study was limited by the proportion of incomplete data regardless of efforts to remediate. However a comparison between our dataset and the NTP register 2004–2006 shows no difference in patient profile [14]. Notwithstanding, the incompleteness highlighted the limitation of the data collection technique. The LA form was concise, the health workers were trained and given incentives to administer them, feedbacks were also given on correctness of completion. Nevertheless, most health staff did not seem to put adequate efforts to administer them properly, which resulted in the lack of completeness of delay data for many of the patients. Seemingly further application of this form must be preceded by efforts to ensure a more enabling working environment. It was also impossible to determine the relative contribution of patient and healthcare provider to the total delay. Evidently, further investigation including measurements of patient delay and health system delay would be necessary to assess the quality of health facilities and barriers to access DOTS facilities. Nonetheless, early diagnosis and treatment (total delay) of TB patients is considered the foundation of TB control. Delay in diagnosis and treatment is associated with greater transmission of infection [15] and poorer outcomes for the patients [16]. Thus, there is a need to elucidate the delay issue in high-burden setting like Indonesia to the possible extend.

Delay between the onset of symptoms of pulmonary tuberculosis and treatment (median 10 weeks) was significantly more common for patients in rural areas. This association between rural setting and delay is in line with findings in other studies [6,17-19]. Factors underlying this association have been documented in these studies. In China, Zhang et al [19] reported that all farmers only seek health care after they failed to treat themselves; and most of them then sought care from less qualified village level health care providers. In Ethiopia, Cambanis et al [6] reported that delays among rural population were associated with transport time, overnight travel, transport cost, having sold personal asset and use of traditional healers. In India, Fochsen et al [18] documented that among rural population the utilisation of public health care services was low, and sputum investigations were rarely performed by both private and public health care providers. Evidently, both financial and structural barriers constrained rural populations across the developing countries from seeking health care.

The data did not reveal a significant association between gender and total delay. Other studies in Asian countries suggested women presenting to the health service with symptoms of TB were diagnosed significantly more slowly than were men [20-22]. The only gender-associated observation from the dataset is that slightly more men than women were screened for TB. This imbalance may be due to either failure of women to seek help within the health system or gender bias during the diagnosis process. Further studies are required to elucidate the reasons for this imbalance. Notwithstanding, the ratio between male and female in our study was rather low (1.34:1). The study in Vietnam [19] for instance revealed a male to female ratio of 2.5:1. Thus, there is a possibility that the magnitude of gender bias within the system is less than other countries or women in our setting have a similar infection risk as men.

This study indicates that the public health centres are the most common first choice of care for tuberculosis patients in the rural districts of Yogyakarta province with 38.3% of individuals presenting to a health centre first. The use of private practitioners as first choice provider (31.1%) however was significantly higher than in the urban areas and among rural population ranked second to the health centre as first choice provider. The presence of private nurse/midwives practices in these rural areas is substantial. Even though these private practices in general are less accessible financially compared to health centres, they are more accessible in geographical terms as well as in regard to working hours. The presence of traditional healers, which are prominent in rural areas across developing countries, have been suggested to contribute to observed delay [23]. A study conducted in Lima, Peru suggested that such an appreciable effect of the use of traditional remedies on TB diagnostic delay is not present in urban population [24]. Our data does not permit affirmation of these observations as we relied on brief structured interviews performed on patients at health facility by health staff. A population based behaviour survey on a randomized sample could better capture a wider range of variables, including the role of traditional healers. This however requires substantial resources, hence should be reserved for a larger evaluation initiative as opposed to what could potentially be generated from a more routine data collection as we have demonstrated.

## Conclusion

In conclusion, this study highlights that an area of vital importance in aiming to make progress toward achieving TB control targets in Indonesia is improving access to DOTS services in rural areas. Recent advocacy have tried to raise attention toward the newly emerging challenge of TB in urban settings. Our data suggests that, while embracing new challenges is indeed a necessity, those who are concerned with TB control simply could not lose sight of the still unfinished business of TB in rural areas.

## Competing interests

The authors declare that they have no competing interests.

## Authors' contributions

YM, BS and AU made substantial contributions to conception and design and acquisition of data. YM and AU made substantial contribution to analysis and interpretation of data. YM, BS and AU have been involved in drafting the manuscript. YM and AU have contributed to revising the manuscript critically for important intellectual content. YM, BS and AU have given final approval of the version to be published.

## Pre-publication history

The pre-publication history for this paper can be accessed here:


